# Physical Activity Modulates the Effect of Cognitive Control on Episodic Memory

**DOI:** 10.3389/fpsyg.2020.00696

**Published:** 2020-04-23

**Authors:** Donglin Shi, Fengji Geng, Yuzheng Hu, Qinmei Xu

**Affiliations:** ^1^Department of Curriculum and Learning Sciences, Zhejiang University, Hangzhou, China; ^2^The Children’s Hospital, Zhejiang University School of Medicine, Zhejiang University, Hangzhou, China; ^3^Department of Psychology and Behavioral Sciences, Zhejiang University, Hangzhou, China

**Keywords:** physical activity, episodic memory, cognitive control, item memory, source memory

## Abstract

Physical activity may improve cognitive control and episodic memory. Cognitive control could exert positive or negative influences on episodic memory. This study aimed to test whether physical activity modulated the effect of proactive and reactive control on episodic memory. Participants reported their physical activity in the past week, encoded episodic memory incidentally in proactive and reactive conditions, and subsequently retrieved their memories of items and sources. Subsequent item memory was better when items were encoded in proactive vs. reactive condition. Smaller condition difference in subsequent item memory was related to better cognitive control ability. Cognitive control completely mediated the relation between physical activity and the condition difference in subsequent item memory. Additionally, condition difference in subsequent source memory was negatively related to cognitive control. After controlling for cognitive control, greater physical activity was positively related to the difference in subsequent source memory between proactive and reactive conditions. Altogether, the findings suggested that physical activity modulated the effect of proactive and reactive control on subsequent item memory through improving cognitive control ability, but it was independent of cognitive control for subsequent source memory.

## Introduction

Physical activity has received much attention as a way to prevent cognitive declines due to the close relations between physical activity and cognitive abilities (Hillman et al., 2008; Lambourne and Tomporowski, 2010; Donnelly et al., 2016). For example, physical activity has been found to be positively related to information processing efficiency, attention, memory, and cognitive control abilities (Buck et al., 2008; Chaddock et al., 2011; Scudder et al., 2015; Drollette et al., 2016). Generally, these previous studies have focused on the relations between physical activity and the functions of a single cognitive system. In fact, different cognitive systems interact with each other in our daily life (Morris et al., 2005; Dudukovic and Kuhl, 2017). However, few previous studies have investigated whether physical activity affects the interactions between cognitive systems. The current study sought to fill this gap by investigating whether physical activity modulated the effect of cognitive control on episodic memory.

Cognitive control refers to the ability to regulate, coordinate, and sequence thoughts and actions in accordance with internally maintained goals according to the dual mechanisms of cognitive control (DMC) theory (Braver, 2012). DMC theory suggests that we shift between proactive and reactive control states in our daily life (Braver, 2012). Proactive control refers to the cue-driven and top-down process, which needs to maintain goals before the occurrence of upcoming stimuli. Reactive control refers to the probe-driven and bottom-up process, which relies upon the detection and resolution of interference after its onset (Braver et al., 2007). Compared with reactive control, proactive control poses a greater demand on brain resources, resulting in extended activation of neural networks (e.g., lateral PFC), and greater metabolic consumption (Botvinick et al., 2001; Braver et al., 2007).

Reduced cognitive control has been reported to benefit episodic memory (Chiu and Egner, 2015b; Amer et al., 2016). This memory type refers to the memories of events and related contextual details (Tulving, 2002; Riggins, 2014; Xue, 2018). The negative effect of cognitive control on episodic memory has been interpreted as the results of common resource competition (Chiu and Egner, 2015a, b). For example, subsequent memories were impaired for stimuli that were encoded in trials requiring response inhibition than those that were encoded in trials without such requirement (Chiu and Egner, 2015b). Subsequently, this finding was supported by fMRI results indicating that trials engaging response inhibition would reduce activity in ventrolateral prefrontal cortex (PFC) that was involved in the encoding of stimuli (Chiu and Egner, 2015a, b). Therefore, as a result of the resources recruited by cognitive control, the resources available for encoding new information may be decreased.

Alternatively, cognitive control has been suggested to promote episodic memory due to attentional modulations (Krebs et al., 2013; Dudukovic and Kuhl, 2017). Top-down attention activated in proactive condition may help encoding episodic memory compared to bottom-up attention induced by reactive control (Dudukovic and Kuhl, 2017). For example, previous study indicated that subsequent memories were better for the stimuli encoded in the condition involving top-down attention than in the one involving bottom-up attention (Dudukovic and Kuhl, 2017). fMRI results indicated that dorsal parietal cortex engaged during top-down attention was recruited during the successful encoding of episodic memories, whereas ventral parietal cortex engaged during bottom-up attention was more active during encoding failure (Dudukovic and Kuhl, 2017). Additionally, functional connectivity analyses indicated that stronger connectivity between dorsal parietal cortex and perceptual regions was associated with a higher likelihood that the attended stimuli would be remembered, whereas such relation was inversed for ventral parietal cortex (Dudukovic and Kuhl, 2017). Therefore, cognitive control could exert positive or negative effects on episodic memory and such effects may vary dependent upon the attentional status modulated by cognitive control.

Cognitive control ability can be improved by increased physical activity (Hillman et al., 2009b; Chaddock-Heyman et al., 2014; Bidzan-Bluma and Lipowska, 2018; de Greeff et al., 2018). For example, meta-analysis study indicated that longitudinal physical activity program has positive effect on cognitive control ability (de Greeff et al., 2018). In addition, fitness, as a measure closely related to physical activity, has been reported to be related to the flexible use of cognitive control strategies according to task demands (Kamijo et al., 2016). Specifically, ERPs measures showed that fitness was positively related to brain response (indicated by greater amplitude of ERP components) during response preparation, suggesting stronger proactive inhibition and early allocation of greater attention resources to process relevant stimuli (Kamijo et al., 2016). Collectively, previous studies suggested that physical activity might improve cognitive control abilities.

Physical activity has also been associated with episodic memory. For example, researchers provided 6-month low or medium intervention on physical activity to elderly individuals and found that after intervention, the total increases in physical activity were positively related to the improvement in episodic memory (Ruscheweyh et al., 2011). Additionally, the ability in recognizing the items encoded relationally has been found to be positively related to aerobic fitness in preadolescents and old adults (Chaddock et al., 2011; Hayes et al., 2015).

The effect of physical activity on cognitive control and episodic memory might be partly driven by the changes in brain structures and functions. The most studied brain regions included prefrontal cortex (PFC) and hippocampus (Chaddock-Heyman et al., 2013; Duzel et al., 2016). For example, after 6-month physical activity intervention to elderly population, the total increase in physical activity was positively related to the increases in PFC volume (Duzel et al., 2016). Aerobic fitness that is closely related to physical activity has been found to be positively associated with preadolescents’ hippocampal volume (Chaddock et al., 2010). In addition to the sizes of PFC and hippocampus, previous studies have also found that physical activity was related to brain functions (Colcombe et al., 2004; Voss et al., 2010). For example, high-fit or aerobically trained participants showed greater PFC activity than low-fit or non-aerobic control ones (Colcombe et al., 2004). Studies in animals indicated that exercise led to neurogenesis in the dentate gyrus of the hippocampus (Voss et al., 2010).

The current study aimed to test whether physical activity modulated the effect of cognitive control on episodic memory. Specifically, we would compare the differences in subsequent memories for items and sources (i.e., subsequent item memory, subsequent source memory) that were encoded in proactive vs. reactive condition. Then, we would test whether such condition differences in subsequent memories were related to individual variation in cognitive control ability and physical activity. Besides, we would test whether the relations of physical activity to the condition differences in subsequent memories were modulated or mediated by individual cognitive control ability. As physical activity was associated with cognitive control that could exert positive or negative influences on episodic memory, we predicted that physical activity would modulate the differences in subsequent memories for items and sources that were encoded in proactive vs. reactive condition. Besides, we predicted that individual differences in cognitive control ability would modulate or mediate the relations between physical activity and the condition differences in subsequent memories for items and sources.

## Materials and Methods

### Participants

We recruited a total of 51 college students from Zhejiang University (mean age = 22.2 years, SD = 1.93, 32 female). A total of 19 subjects were not included in final analyses because three did not learn how to perform the tasks and 16 reported that they could not remember the duration or frequency of their physical activity during last seven days. Self-report indicated that all subjects were healthy without color-blindness, adverse health conditions, physical incapacities, or neurological disorders. This study was approved by the IRB of Zhejiang University and participants signed the consent form indicating they were voluntarily to participate in the study.

### Physical Activity Questionnaires

Participants completed the short version of International Physical Activity Questionnaire (IPAQ) to measure their physical activity level during last seven days (Hagströmer et al., 2006). The items in the questionnaire were structured to measure the volume of vigorous-intensity activity, moderate-intensity activity, and walking per week. These activities were weighted by their energy requirements defined in MET (Metabolic Equivalent Task) to generate a score in MET-minutes, which is calculated by multiplying the MET score of an activity by the minutes performed (walking = 3.3 METs, moderate activity = 4.0 METs, and vigorous activity = 8.0 METs). Total physical activity (MET-min/week) was calculated by the summation of walking, moderate, and vigorous activity in MET-minutes over a week. This summation score was used as continuous variable to measure physical activity level in the current study.

### Behavioral Task

Participants completed a computer-based incidental learning task to measure both cognitive control and episodic memory, designed based on previous studies (Chevalier et al., 2015; Geng et al., 2019). This task was comprised of encoding and retrieval phases, both coded and presented using OpenSesame (Mathôt et al., 2012). During encoding, there were two sessions and each session engaged a specific cartoon character (Winnie or Donald) presented as background.

**FIGURE 1:**
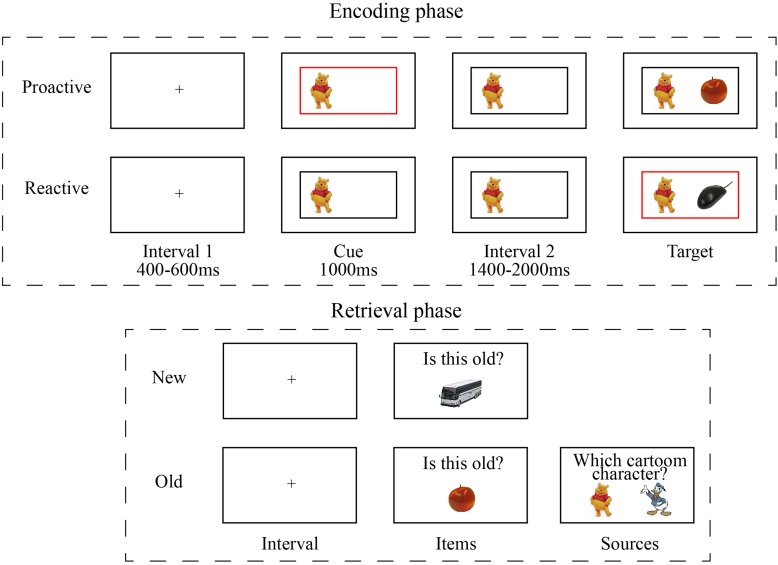
Design of the incidental learning task.

In each session, participants completed a proactive control block and a reactive control block. The two control conditions were manipulated by presenting informative border at different timing (Figure 1). In proactive control condition, the informative border (red or green) was present with the cue before the onset of target picture, whereas in reactive control condition, non-informative (black) border was presented with the cue and the informative border was simultaneously presented with target picture. In each block, the color of the informative border and the background cartoon character determined the rules for judging target pictures. For example, the combination of “red border” and “Winnie” indicated that the rule was to judge whether the target was an animal picture, whereas the combination of “green border” and “Winnie” implied to judge whether the target was a food picture. The rules were counterbalanced between subjects.

Each block included 40 trials and lasted about 4.5 min. In each trial, the target picture (e.g., apple) and cartoon character (i.e., Winnie or Donald) served as items and sources, respectively. The participants were instructed to respond to the target picture as quickly and accurately as possible. Differences in reaction times between proactive and reactive conditions were used to define cognitive control.

After encoding task, participants took 10 to 15 min to fill out several questionnaires before performing retrieval task. During retrieval phase, there were 210 pictures (160 old pictures and 50 new pictures) presented and participants needed to judge whether a picture was old (seen during encoding) or new. If a picture was judged as “old”, participants was promoted to judge which cartoon character was presented with the “old” item. The proportion of old items that were correctly recognized was defined as item memory. Among the recognized “old” items, the proportion for which cartoon characters had been correctly recognized was defined as source memory.

### Statistical Analysis

Repeated measures ANOVA model was used to compare the differences in reaction times or accuracy between proactive and reactive conditions during encoding phase. We also used this model to test the differences in subsequent memory of items and sources encoded in proactive vs. reactive condition. In these models, condition was included as within subject variable (proactive vs. reactive). Additionally, physical activity or/and cognitive control were included as covariates. An SPSS-based macro developed by Hayes (2017) was used to test how individual differences in cognitive control abilities mediated the relations between physical activity level and the differences in subsequent item or source memory between proactive and reactive control conditions.

## Results

During encoding, participants responded faster in proactive vs. reactive control condition [*F*(1,30) = 35.36, *p* < 0.001] with no differences in accuracy between the two conditions (*p* = 0.529), Table 1. Therefore, the manipulation of proactive and reactive control conditions was effective in the incidental learning task. During retrieval, all participants included in final analyses showed greater hit rates (mean = 0.58, SD = 0.10) than false alarm rates (mean = 0.15, SD = 0.10), suggesting that they did not perform the retrieval task by just guessing. Additionally, subsequent item memory was greater in proactive vs. reactive condition [*F*(1,30) = 7.21, *p* = 0.012], and the condition difference was not significant for subsequent source memory (*p* = 0.430), Table 1.

**TABLE 1:** Behavioral results (mean, SD) during Encoding and Retrieval phases in the incidental learning task.

Condition	Encoding		Retrieval	
		
	RTs (ms)	ACC (%)	Item	Source
			memory (%)	memory (%)
Proactive	611.04 (139.09)	92.3 (7.77)	59.69 (12.20)	53.85 (9.00)
Reactive	800.02 (230.51)	92.8 (6.46)	56.21 (10.07)	55.33 (6.32)

### Physical Activity and Cognitive Control

**FIGURE 2:**
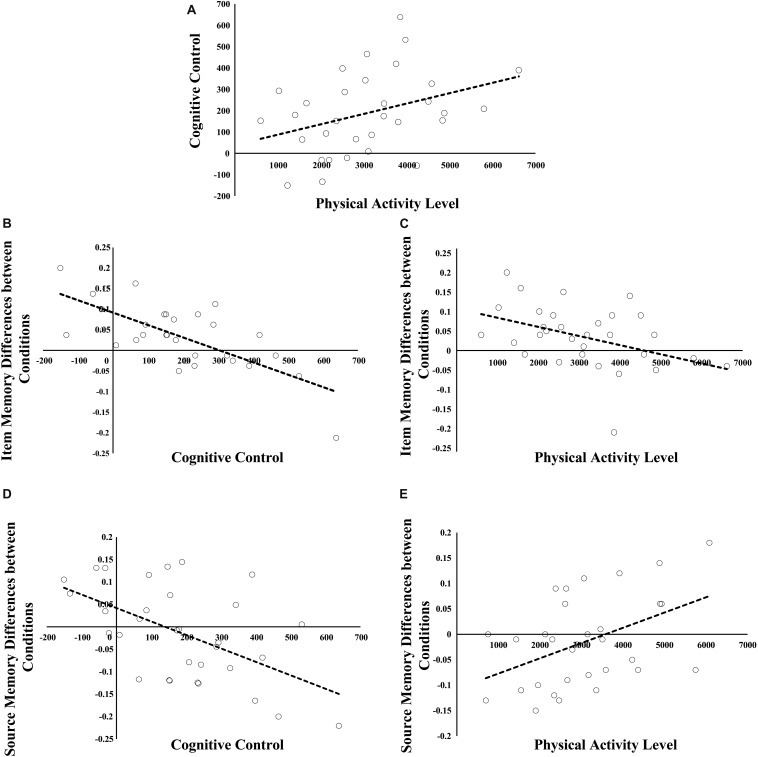
Relations among physical activity, cognitive control, and the differences in subsequent item and source memory between proactive and reactive conditions. **(A)** represents the relation between physical activity and cognitive control. **(B,C)** stand for the relations of cognitive control and physical activity to the condition differences in subsequent item memory. **(D,E)** show the relations of cognitive control and physical activity to the condition differences in subsequent source memory.

There was positive relation between physical activity and cognitive control (*r* = −0.359, *p* = 0.044; see Figure 2A) as suggested by the significant interaction between condition and physical activity [*F*(1,30) = 4.44, *p* = 0.044]. The interaction suggested that greater physical activity was related to even faster response in proactive vs. reactive condition (the differences in RTs between proactive and reactive conditions were used to measure individual cognitive control ability). Correlation analyses indicated that there was a trending positive relation between physical activity and mean RTs in reactive condition (*r* = 0.34, *p* = 0.055), whereas such relation was not significant in proactive condition (*p* > 0.60).

### Physical Activity, Cognitive Control and Subsequent Item Memory

Greater physical activity was related to smaller difference in subsequent item memory between proactive and reactive conditions (*r* = −0.422, *p* = 0.016; see Figure 2B). The finding was indicated by the significant interaction between condition and physical activity when only physical activity was included as covariate [*F*(1,30) = 6.49, *p* = 0.016]. The interaction indicated that the condition difference in subsequent item memory was negatively related to physical activity (after removing the extreme value, the correlation was still significant, *r* = −0.446, *p* = 0.012).

To interpret the interaction between condition and physical activity, we tested whether physical activity was related to subsequent item memory in proactive and reactive conditions, respectively. There was a trending positive relationship between physical activity and subsequent item memory in reactive control condition (*r* = 0.31, *p* = 0.08), whereas the relation was not significant in proactive control condition (*p* > 0.90). Additionally, according to median physical activity level, we separated children into low and high physical activity groups. In the low group, subsequent item memory was better in proactive vs. reactive control condition [*F*(1,15) = 15.64, *p* = 0.001], whereas the difference was not significant in high physical activity group (*p* > 0.08).

Furthermore, greater cognitive control was related to smaller difference in subsequent item memory between proactive and reactive conditions (*r* = 0.718, *p* < 0.001; see Figure 2C). This finding was supported by the significant interaction between condition and cognitive control when only cognitive control was included as covariate [*F*(1,30) = 31.91, *p* < 0.001]. To interpret this interaction, we tested whether cognitive control was related to subsequent item memory in proactive and reactive control conditions. Cognitive control was positively related to subsequent item memory in reactive condition (*r* = 0.48, *p* = 0.006), whereas such relation was not significant in proactive condition (*p* > 0.60). In addition, according to median value of cognitive control, we separated children to low and high cognitive control groups. In the low group, subsequent item memory was better in proactive vs. reactive condition [*F*(1,15) = 34.49, *p* < 0.001], whereas the difference was not significant in high cognitive control group (*p* > 0.53).

**FIGURE 3:**
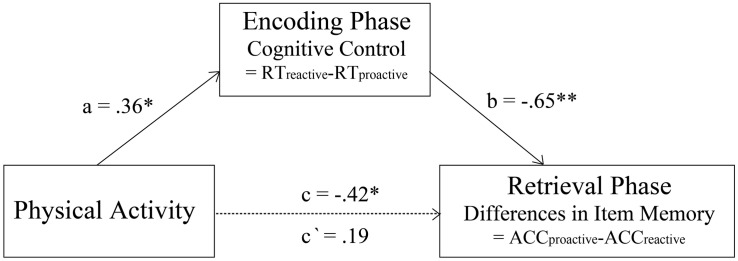
Complete mediation effect of cognitive control on the relation between physical activity and the condition difference in subsequent item memory (*indicates *p* < 0.05, **indicates *p* < 0.01).

Since physical activity, cognitive control, and condition differences in subsequent item memory were related to each other, we conducted mediation analyses to test whether cognitive control mediated the relation between physical activity and the condition differences in subsequent item memory. The result indicated that the total indirect effect (Figure 3; *b* = −0.65, *p* < 0.001) with a 95% bootstrap CI of −0.53 to −0.05, which did not include zero. Therefore, cognitive control completely mediated the relation between physical activity and the differences in subsequent item memory between proactive and reactive conditions. This result indicated that the impact of physical activity on the differences in subsequent item memory between proactive and reactive conditions occurred completely through cognitive control.

### Physical Activity, Cognitive Control and Subsequent Source Memory

When physical activity was included as covariate, repeated measures ANOVA model indicated that there was no significant effect involving condition or physical activity. In contrast, when cognitive control was included, there was a significant interaction between condition and cognitive control [*F*(1,30) = 12.56, *p* = 0.001], indicating that cognitive control was negatively related to the differences in subsequent source memory between proactive and reactive conditions (*r* = −0.54, *p* = 0.001; see Figure 2D). To interpret the interaction better, we tested the relations of cognitive control to subsequent source memory in proactive and reactive condition, respectively. The results indicated that cognitive control was negatively related to subsequent source memory in proactive condition and such relation was inversed in reactive condition (*r* = −0.36, *p* = 0.04; *r* = 0.39, *p* = 0.03).

**FIGURE 4:**
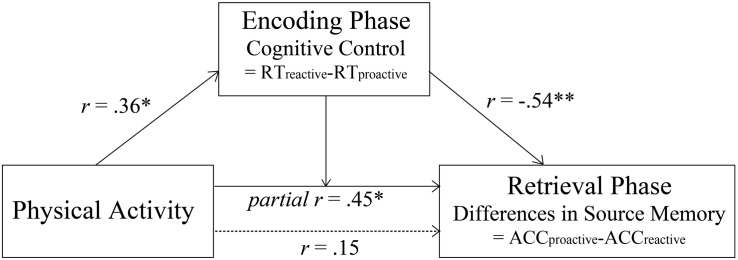
Significant relation between physical activity and the condition difference in subsequent source memory after controlling cognitive control (*indicates *p* < 0.05, **indicates *p* < 0.01).

Then, we included both physical activity and cognitive control as covariates in the model in order to test whether there was interaction between physical activity and condition after controlling for cognitive control. The results indicated that there was significant interaction between condition and physical activity [*F*(1,30) = 7.17, *p* = 0.012], suggesting that physical activity was positively related to the condition differences in subsequent source memory between proactive and reactive conditions after controlling for cognitive control (*r* = 0.45, *p* = 0.012; see Figures 2E, 4). To interpret the interaction, we calculated the partial correlations between physical activity and subsequent source memory in proactive and reactive control conditions, respectively. There was a trending positive correlation between physical activity and subsequent source memory in proactive control condition (*r* = 0.35, *p* = 0.051), whereas such relation was not significant in reactive control condition (*p* > 0.10). Therefore, physical activity may affect the differences in subsequent source memory between proactive and reactive control conditions independent of individual differences in cognitive control ability.

## Discussion

This study sought to test whether physical activity modulated the effect of proactive and reactive control on episodic memory. As expected, physical activity was negatively related to the differences in subsequent item memory between proactive and reactive conditions and such relation was completely mediated by individual differences in cognitive control ability. Additionally, cognitive control was negatively related to the condition differences in subsequent source memory. Inconsistent with our expectation, only after controlling for individual variation in cognitive control, physical activity was positively related to the differences in subsequent source memory between proactive and reactive conditions. Therefore, physical activity might affect the differences in subsequent item memory between proactive and reactive conditions through improving individual cognitive control ability, but the effect of physical activity on subsequent source memory was independent of cognitive control ability.

### Relations Between Physical Activity and Cognitive Control

This study supported previous findings by revealing the positive relations between physical activity and individual cognitive control ability (Hillman et al., 2009a; Chaddock-Heyman et al., 2014; Bidzan-Bluma and Lipowska, 2018; de Greeff et al., 2018). The positive relation may reflect that people with greater physical activity are better at using valid cues to prepare their responses to incoming targets than the ones with lower physical activity. This interpretation is consistent with previous neuroelectric studies suggesting that children with higher fitness are better at allocating attentional resource and have enhanced cognitive flexibility in tasks demanding cognitive control (Hillman et al., 2009a; Pontifex et al., 2011). Additionally, there was a trending positive relation between physical activity and the reaction times in reactive condition, but the relation was not significant in proactive condition. This result may indicate that when there are no external cues to guide their response, participants with higher physical activity can main their behavioral goals more efficiently than the ones with lower physical activity. This finding lends support to the argument that physical activity is more related to the cognitive functions that require vast amount of cognitive control (Kramer et al., 1999; Hillman et al., 2006). Finally, previous studies have suggested that physical activity has positive influences on the functions of brain regions (e.g., PFC), which are closely related to cognitive control (Kramer and Erickson, 2007; Berchicci et al., 2013; Chaddock-Heyman et al., 2013). Such changes in brain functions may be the neural mechanism underlying the positive relation between physical activity and cognitive control in the current study.

### Relations of Physical Activity and Cognitive Control to Subsequent Item Memory

The findings of this study suggested that individual variation in physical activity and cognitive control were related to the differences in subsequent item memory between proactive and reactive conditions. Adults with lower physical activity or cognitive control ability showed better subsequent item memory in proactive vs. reactive condition, whereas such difference was not significant in the group with greater physical activity or cognitive control ability. Furthermore, through mediation analyses, we found that the relation between physical activity and the condition differences in subsequent item memory was completely mediated by individual variation in cognitive control ability. Therefore, physical activity may affect the differences in subsequent item memory between proactive and reactive conditions completely through improving cognitive control ability.

The mediation effect may be related to the differences in how attention is modulated in proactive and reactive conditions. In these two conditions, proactive and reactive cues have been suggested to drive top-down and bottom-up attention, respectively (Braver, 2012). Top-down attention has been suggested to foster the incidental encoding of items within the focus of attention (Uncapher et al., 2011), which might be the reason that subsequent item memory was better in proactive vs. reactive condition in the group lower on cognitive control ability. In contrast, in the group higher on cognitive control, subsequent item memory was not different between proactive and reactive conditions. Besides, there was a trending positive relation between cognitive control and subsequent memory for items encoded in reactive condition. We speculated that for the individuals with greater cognitive control ability, although cues in reactive condition did not externally drive top-down attention, such attention mode might be voluntarily engaged with the aim to making response as fast as possible in the reactive condition. As a result, the individuals with greater cognitive control ability may have improved subsequent item memory in reactive condition, which may reduce the differences in subsequent item memory between proactive and reactive conditions. The reduced differences might be partially related to physical activity because enhanced physical activity has been suggested to improve cognitive control ability that can drive the engagement of top-down attention in reactive condition.

### Relations of Physical Activity and Cognitive Control to Subsequent Source Memory

There was no difference in subsequent source memory between proactive and reactive conditions. However, the condition differences in subsequent source memory was negatively related to cognitive control ability, indicating that greater cognitive control ability was associated with smaller subsequent source memory in proactive vs. reactive condition. Such relation can be interpreted according to the cognitive resource competition theory (Kahneman, 1973; Chiu and Egner, 2015b). For the individuals with greater cognitive control ability, the top-down attention elicited by proactive cues may result in more cognitive resources invested into cognitive control and the incidental encoding of items within the focus of attention, but less resources remained for encoding the sources out the focus of attention. This mechanism might underlie the negative relation between cognitive control and subsequent source memory in proactive condition. In contrast, the cues in reactive condition did not drive top-down attention but the targets elicited bottom-up attention to process the rules and make responses. During this process, the individuals with better cognitive control ability might encode both the items and the accompanied sources more deeply, which might lead to the positive relation between cognitive control and subsequent source memory in reactive condition.

Additionally, after controlling for individual variation in cognitive ability, physical activity was positively related to the differences in subsequent source memory between proactive and reactive conditions. Especially, there was a trending positive relation between physical activity and subsequent memory for sources encoded in proactive condition. Therefore, increased physical activity may benefit subsequent source memory in proactive condition and as a result, the condition differences in subsequent source memory between proactive and reactive conditions were improved. This finding can also be interpreted according to the common resource competition theory (Kahneman, 1973; Chiu and Egner, 2015b). Previous studies have suggested that physical activity can improve the functions of brain structures related to various cognitive process, which might finally lead to increased common resources (Ploughman, 2008; Zoeller, 2010; Erickson et al., 2015). Therefore, intensified physical activity may not only increase cognitive resources for encoding contextual information but also reduce the resource competition between cognitive control and encoding in proactive condition in the incidental learning task used by the current study. Such effect of physical activity was independent of individual cognitive control ability.

### Strengths and Limitations

The strengths of the current study included that we investigated the impact of physical activity on the interactions between cognitive control (proactive vs. reactive control) and episodic memory (item memory vs. source memory), rather than just a single cognitive system. Despite the strengths, there are limitations that warrant mention. First, the limited sample size may affect statistical power. For example, some results only showed trending significance. Additionally, although IPAQ has been used widely, previous studies have reported the differences in physical activity measured by the accelerometer and IPAQ (Randy et al., 2003; Shephard, 2003). Future studies need to combine multiple methods to define physical activity. Finally, the current study only focused on the associations between physical activity and cognitive functions, and such associations could be interfered by other confounding factors. Future research could construct the causal relationships between physical activity and cognitive functions by manipulating physical activity level experimentally.

## Conclusion and Future Directions

To summarize, this study indicated that physical activity modulated the differences in episodic memory between different cognitive control conditions. Specifically, physical activity affected the differences in subsequent item memory between proactive and reactive control conditions completely through modulating individual differences in cognitive control ability. In contrast, physical activity modulated the effect of proactive and reactive control on subsequent source memory independent of individual differences in cognitive control. The findings highlight that physical activity modulates the interactions between cognitive control and episodic memory systems and such modulation varies among different cognitive systems. These implications are important to consider in terms of intervening cognitive declines through increasing physical activity. Future research still needs to identify the neural mechanism underlying the impact of physical activity on the interactions between cognitive systems.

## Data Availability Statement

The datasets generated for this study are available on request to the corresponding author.

## Ethics Statement

The studies involving human participants were reviewed and approved by the Zhejiang University. The patients/participants provided their written informed consent to participate in this study.

## Author Contributions

DS drafted the initial manuscript, carried out the data analysis, interpreted the results, and critically revised the manuscript for important intellectual content. FG conceived and designed the study, contributed to acquisition, analysis, and interpretation of data, drafted, reviewed, and critically revised the manuscript for important intellectual content. YH contributed to conception, data collection, and critically revised the manuscript for important intellectual content. QX contributed to conception and critically revised the manuscript for important intellectual content. All authors approved the final manuscript as submitted.

## Conflict of Interest

The authors declare that the research was conducted in the absence of any commercial or financial relationships that could be construed as a potential conflict of interest.
